# Cyanobacterial PHA Production—Review of Recent Advances and a Summary of Three Years’ Working Experience Running a Pilot Plant

**DOI:** 10.3390/bioengineering4020026

**Published:** 2017-03-28

**Authors:** Clemens Troschl, Katharina Meixner, Bernhard Drosg

**Affiliations:** 1 Institute of Environmental Biotechnology, Department of Agrobiotechnology, IFA-Tulln, University of Natural Resources and Life Sciences, Vienna, Tulln 3430, Austria; clemens.troschl@boku.ac.at; 2 Bioenergy2020+ GmbH, Tulln 3430, Austria; bernhard.drosg@bioenergy2020.eu

**Keywords:** cyanobacteria, polyhydroxyalkanoates, CO_2_ mitigation, flue gas utilization, photobioreactor

## Abstract

Cyanobacteria, as photoautotrophic organisms, provide the opportunity to convert CO_2_ to biomass with light as the sole energy source. Like many other prokaryotes, especially under nutrient deprivation, most cyanobacteria are able to produce polyhydroxyalkanoates (PHAs) as intracellular energy and carbon storage compounds. In contrast to heterotrophic PHA producers, photoautotrophic cyanobacteria do not consume sugars and, therefore, do not depend on agricultural crops, which makes them a green alternative production system. This review summarizes the recent advances in cyanobacterial PHA production. Furthermore, this study reports the working experience with different strains and cultivating conditions in a 200 L pilot plant. The tubular photobioreactor was built at the coal power plant in Dürnrohr, Austria in 2013 for direct utilization of flue gases. The main challenges were the selection of robust production strains, process optimization, and automation, as well as the CO_2_ availability.

## 1. Introduction

Polyhydroxyalkanoates (PHAs) are considered as one of the most promising bioplastics. Their mechanical properties are similar to polypropylene and they can be processed in a similar way, including extrusion, injection molding, or fiber spinning [1]. One of the major advantages of PHAs are their biodegradability. They are degraded relatively rapidly by soil organisms, allowing easy composting of PHA waste material [2].

Currently, PHA is produced in large fermenters by heterotrophic bacteria, like *Cupriavidus necator* or recombinant *Escherichia coli* [3]. For these fermentation processes large amounts of organic carbon sources like glucose are necessary, accounting for approximately 50% of the total production costs [4]. An alternative way of producing PHA is the use of prokaryotic algae, better known as cyanobacteria. As part of the phytoplankton, they are global primary biomass producers using light as the sole energy source to bind atmospheric CO_2_ [5]. Burning of fossil fuels has increased the atmospheric CO_2_ concentration from approximately 300 ppm in 1900 to over 400 ppm today. The latest report of the intergovernmental panel on climate change (IPCC) clearly indicates anthropogenic CO_2_ emissions as the main driver for climate change [6]. Given these facts, cultivation of cyanobacteria for PHA production could be a more sustainable way of producing bioplastics.

## 2. Cyanobacteria and Cyanobacterial Energy and Carbon Storage Compounds

**Figure 1:**
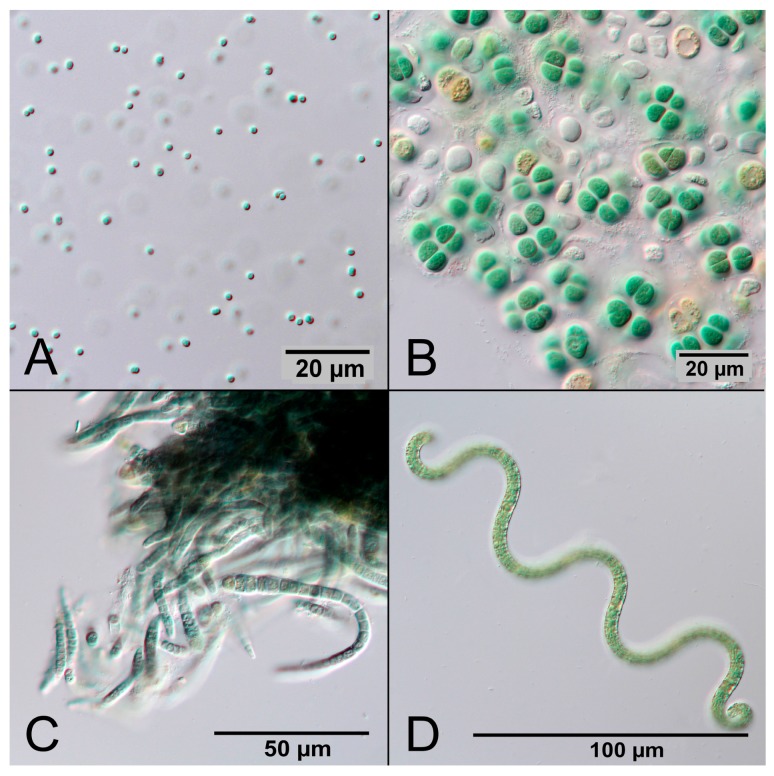
Microscopic photographs of different cyanobacterial species made in DIC (differential interference contrast). (**A**) *Synechocystis* sp.; (**B**) *Cyanosarcina* sp.; (**C**) *Calothrix* sp.; and (**D**) *Arthrospira* sp.

Cyanobacteria are Gram-negative prokaryotes that perform oxygenic photosynthesis. They are abundant in illuminated aquatic ecosystems and contribute significantly to the world carbon and oxygen cycle [7]. According to current evidence, oxygen was nearly absent in the Earth’s early atmosphere until 2.4 billion years ago [8]. Due to oxygenic photosynthesis of early cyanobacteria the CO_2_-rich atmosphere gradually turned into an oxygen-rich atmosphere, providing the conditions for multicellular life [9,10]. Today there are an estimated 6000 species of cyanobacteria with great diversity, for example ranging in size from the 1 µm small unicellular *Synechocystis* sp. to the several millimeter-long multicellular filaments of *Oscillatoria* sp. [11]. The common feature of cyanobacteria is the presence of the pigment phycocyanin, which gives them their typical blue-green color. Figure 1 shows photographs of four different cyanobacterial species.

### 2.1. Cyanobacteria–Microalgae or Not?

For more than a century, cyanobacteria were considered as an algal group under the general name “blue-green algae”. They were classified under the International Code of Botanical Nomenclature, nowadays called the International Code of Nomenclature for Algae, Fungi, and Plants (ICN). In 1980 the International Code of Nomenclature of Bacteria, nowadays called the International Code of Nomenclature of Prokaryotes (ICNP), was established. Stanier, one of the leading cyanobacteria researchers at that time, proposed the inclusion of cyanobacteria in the ICNP [12]. Nevertheless, the ICNP was not consistently applied for cyanobacteria and cyanobacteria are still covered by the ICN as well. The latest preamble of the ICN clarifies, that this code applies to all organisms traditionally treated as algae, fungi, or plants, including cyanobacteria [13]. Today cyanobacteria continue to be covered by both the Botanical Code (ICN) and Prokaryotic Code (ICNP). An effort to reconcile the status of this group of bacteria has been underway for several decades. Although some progress has been made, a final decision has not yet been reached [14]. From a phylogenetic point of view, there is a clear distinction between prokaryotic cyanobacteria and eukaryotic green algae. However, phycologists regard any organism with chlorophyll *a* and a thallus not differentiated into roots, stem, and leaves to be an alga. Therefore, in phycology, the term microalgae refers to both eukaryotic green algae and cyanobacteria, microscopic in size [15].

### 2.2. Cyanobacterial PHA

Polyhydroxyalkanoates (PHAs) can be classified into three groups: short-chain-length-PHA (scl-PHA), medium-chain-length-PHA (mcl-PHA), and long-chain-length-PHA (lcl-PHA). They differ in mechanical and thermal properties [16]. Among the different PHAs, polyhydroxybutyrate (PHB) is by far the most common and the only PHA produced under photoautotrophic conditions reported so far. Other scl-PHAs, like P[3HB-co-3HV], are only produced when adding organic carbon precursors, like valerate, to the medium. No mcl-PHA or lcl-PHA have been reported in cyanobacteria. Therefore, the term PHB is used in this study, if no other specific PHA is described.

PHB is frequently found in cyanobacteria as an energy and carbon storage compound. In the biosphere they often have to cope with unfavourable environmental conditions. One of the most important growth limiting factors is the absence of nutrients. Nitrogen limitation is the most important and best studied trigger for PHB production in cyanobacteria [17,18,19]. Non-diazotrophic strains are not able to bind molecular nitrogen and depend on nitrogen in the form of nitrate or ammonium. Nitrogen-depleted cells cannot synthesize the necessary proteins for reproduction and, therefore, start to accumulate storage compounds like PHB. Another important function of PHB synthesis is to compensate imbalanced metabolic situations, as it acts as an electron sink and delivers new reduction equivalents in the form of NADP^+^ [18,19,20].

The model organism *Synechocystis* PCC6803 is the best-studied cyanobacterium, and its genome was fully sequenced in 1997 [21]. Most of the understanding of cyanobacterial PHB formation was gained by research done with *Synechocystis* PCC6803. Biosynthesis of PHB from the precursor acetyl-CoA takes place in three steps. Acetoacetyl-CoA is produced from two molecules of acetyl-CoA in a Claisen type condensation by β-ketothiolase. Next step is the reduction of acetoacetyl-CoA by the acetoacetyl-CoA reductase to form d-3-hydroxybutyryl-CoA. Ultimately, PHB is formed in a polymerization reaction by the PHA-synthase. The necessary three enzymes are encoded by the four genes phaA (slr1993), phaB (slr1994), phaC (slr1830), and phaE (slr1829). phaA and phaB are organized in one operon encoding for the β-ketothiolase and acetoacetyl-CoA reductase. phaC and phaE are also organized in one operon encoding the two subunits of the type III PHA synthase [22,23].

### 2.3. Cyanobacterial Glycogen

Regarding PHB synthesis, it should be kept in mind that cyanobacteria also produce glycogen as a second carbon and energy storage compound under nitrogen depletion. In fact, the glycogen content is most often higher than the PHB content and varies between 20% and 60% [24,25,26,27,28]. While PHB is produced in 3–8 larger granules, glycogen is stored in many small granules [18,29,30,31,32,33]. Glycogen is synthesized instantly after nitrogen depletion while PHB synthesis is slower [34]. Glycogen is also produced in non-depleted cells with lower content, aiding the cell to cope with short term energy deficits like the day-night cycle. Glycogen deficient mutants were shown to be highly sensitive to day-night cycles [35]. Glycogen synthesis is a highly-conserved feature abundant in all cyanobacterial genomes reported so far [36]. PHB synthesis on the other hand is common in many, but not all, cyanobacteria [37,38]. Glycogen shows similarities to starch in green algae, while PHB synthesis shows some similarities to triacylglycerol (TAG) synthesis in green algae, where TAG synthesis also serves as an electron sink and consumes excess NADPH [39,40].

In a recent study Damrow and colleagues compared PHB-deficient mutants to glycogen-deficient mutants of *Synechocystis* PCC6803. Glycogen-deficient mutants could not switch to a dormant metabolic state and could not recover from nitrogen depletion. Excess carbon was mostly secreted into the medium in the form of 2-oxoglutaric acid and pyruvate, although the PHB content also increased from 8% to 13%. PHB-deficient mutants, on the other hand, behaved very much like the wild-type with the same amount of glycogen accumulation and the same recovery capability. Only double-knockout mutants (glycogen and PHB deficient) were most sensitive and showed a reduced growth rate, signs for a very specific role of PHB in cyanobacteria, which is still not totally clear [41]. The reported studies show that inhibiting glycogen synthesis increases the PHB production, although cells suffer as glycogen plays an important role.

### 2.4. Nitrogen Chlorosis and Photosynthetic Activity

During nitrogen starvation the cells gradually change from a vegetative state to a dormant state. The most obvious feature of this is the change in colour from blue-green to brownish-yellow. This phenomenon is called “nitrogen chlorosis” and was described already at the begin of the 20th century [42]. It is caused by the degradation of the pigments phycocyanin and chlorophyll. When transferring *Synechococcus* PCC7942 to a nitrogen depleted medium, 95% phycocyanin was degraded within 24 h, and after 10 days 95% of the chlorophyll was also degraded [43]. Concomitantly, the activities of the photosystems (PS) I and II decrease strongly and are only about 0.1% compared to vegetative cells [44]. A recent and very interesting study examined the awakening of a dormant *Synechocystis* PCC6803 cell. After the addition of nitrate the yellow culture turned green again within 36 hours. Transmission electron microscopy revealed the rapid degradation of glycogen and PHB. During the first 24 h of this process the cells consumed oxygen. Transcriptome analysis showed the induction of RuBisCO and carboxysom associated RNAs, as well as the photosystem-related RNAs to prepare the cells for vegetative photoautotrophic growth [34]. The results indicate the decrease in photosynthetic activity during nitrogen starvation, which can be considered a significant challenge to photoautotrophic PHB production.

## 3. Different Cyanobacteria as PHA Producers

### 3.1. Synechocystis and Synechococcus

*Synechocystis* and *Synechococcus* are very small (0.5–2 µm) unicellular cyanobacteria abundant in almost all illuminated saltwater and freshwater ecosystems. One of the first detailed descriptions of PHB accumulation in *Synechocystis* PCC6803 was provided by Wu and colleagues. Nitrogen starved cells produced 4.1% PHB of cdw while under-balanced culturing conditions PHB content were under the detection limit [45]. The same strain was examined for PHB production some years later, where 9.5% PHB of cdw were produced under nitrogen limitation. Phosphorous-depleted cells showed 11.2% PHA of cdw. Interestingly, balanced cultivated control cultures already contained 4.5% PHB of cdw. Supplementation of acetate and fructose lead to a PHB content of 38% of cdw [46]. Recently, recombinant *Synechocystis* PCC6803 with overexpression of the native PHA genes were constructed. They showed a PHB content of 26% of cdw under nitrogen-depleted culturing conditions compared to 9.5% of cdw of the wild-type [47]. However, it must be considered that there are legal issues in most countries when cultivating recombinant strains outdoors. In another study the thermophilic strain *Synechococcus* MA19 showed a PHB content of 55% under phosphate-limited culturing conditions. This study was published in 2001 and still reports the highest PHB content under photoautotrophic conditions [48]. Table 1 shows reported PHA values of *Synechocystis* and *Synechococcus*.

### 3.2. Arthrospira (Spirulina)

*Arthrospira* (formally *Spirulina*) is a species of filamentous cyanobacteria that grows naturally in alkaline salt lakes. It has a high protein and vitamin content and is mainly grown as a food supplement. Recent studies have shown its antioxidant, immunomodulatory, and anti-inflammatory activities [49]. From all cyanobacterial species known, only *Arthrospira* sp. is produced at an industrial scale. The main reason for that is the possibility of cultivation in a highly alkaline environment that prevents contamination and enables the maintenance of a stable culture in open ponds. No exact data are available; however, we estimate the world annual production of around 5000–15,000 tons *Arthrospira* sp. dry weight per year [50,51,52,53].

The first description of PHB accumulation in *Arthrospira* was reported by Campbell and colleagues, who described a PHB content of 6% of cdw in a non-optimized mineral medium. Interestingly, the highest PHB content was measured at the end of exponential growth and decreased during stationary phase [54]. In a screening of 23 cyanobacterial strains, *Arthrospira platensis* had the lowest PHB concentration of only 0.5% in a non-optimized medium [37]. In a screening of several *Arthrospira* species the PHB amount never exceeded 1% of cdw in photoautotrophic growth. Addition of sodium acetate led to a PHB amount of 2.5% of cdw [55]. In another experiment *Arthrospira platensis* was grown under phosphate limitation and reached 3.5% PHB of cdw [56]. *Arthrospira subsalsa*, a strain isolated from the Gujarat coast, India, produced 14.7% PHB of cdw under increased salinity [57]. A detailed ultrastructural analysis of *Arthrospira* strain PCC8005 was conducted by Deschoenmaker and colleagues. Under nitrogen depleted conditions PHB granules were more abundant and larger. The nitrogen-starved cells showed an estimated four times higher PHB concentration [27]. Nitrogen starvation was performed in *Arthrospira maxima* and glycogen and PHB content was measured. While the glycogen content increased from around 10% to 60%–70% of cdw, PHB amount remained low at 0.7% of cdw. The addition of sodium acetate increased the PHB amount to 3% of cdw [26].

The performed studies support the idea, that PHB production in *Arthrospira* is highly strain-dependent. Most *Arthrospira* species produce PHB only in amounts of lower than 5%, even with the addition of sodium acetate. *Arthrospira* produces glycogen as storage compound, what has been shown in ultrastructural research, too [27]. Nevertheless, it must be emphasized that *Arthrospira*, at an industrial scale, is still one of the most promising candidates for PHB production with cyanobacteria. Indeed, PHB nanofibers were produced recently from *Arthrospira* PHB and showed highly favourable properties [58,59]. The biggest challenge for further research is to increase the relatively low PHB content of *Arthrospira*. Table 2 shows reported PHA values of *Arthrospira*.

### 3.3. Nostoc

*Nostoc* is a group of filamentous cyanobacteria very common in terrestrial and aquatic habitats. They are capable of fixing atmospheric nitrogen with specialized heterocysts and are suspected to maintain soil fertility in rice fields due to nitrogen fixation [60]. Ge-Xian-Mi, an edible *Nostoc* species, forms spherical colonies that have been collected in China for centuries [61]. The first reports found for PHB production in *Nosctoc muscorum* are from 2005, when Sharma and Mallick showed that *Nostoc muscroum* produced 8.6% PHB of cdw under phosphate and nitrogen limitation during the stationary phase. PHB content could be boosted to 35% of cdw with 0.2% acetate and seven days dark incubation [62]. Limited gas exchange and supply with 0.4% acetate increased the PHB content to 40% [63]. *Nostoc muscorum* was grown photoautotrophically without combined nitrogen sources and four days of phosphate deficiency increased PHB content from 4% to 22% [56]. The co-polymer P[3HB-co-3HV] could be produced by *Nostoc* in a propionate- and valerate-supplied medium. The 3HV fraction ranged from 10–40 mol% and showed desirable properties in terms of flexibility and toughness. Nitrogen and phosphate depletion led to a PHA content of 58%–60% of cdw, however, the total cdw did not exceed 1 g/L [64]. Further process optimization led to a PHA productivity of 110 mg/L/d and a P[3HB-co-3HV] content of 78% of cdw, the highest yield in heterotrophic grown cyanobacteria reported so far [65]. Recently, poultry litter was used for cultivation of *Nostoc muscorum agardh*. The poultry litter contained phosphate, ammonium, nitrate, and nitrite as nutrients for cyanobacterial growth. Optimized conditions, which included the addition of acetate, glucose, valerate, and CO_2_-enriched air, led to a P[3HB-co-3HV] content of 70% cdw. However, total cdw remained relatively low at 0.68 g/L [66].

The reported studies show that PHB content in *Nostoc* can be significantly increased with organic carbon sources, especially in the form of acetate. However, those organic carbon sources lead to heterotrophic growth and may suppress CO_2_ uptake by the cells, which is the most important argument for using cyanobacteria as PHA producers. All of the reported experiments of *Nostoc* were performed in shaking flasks or small reactors under sterile conditions. In mass cultivation *Nostoc* would have to be cultivated under non-sterile conditions and organic carbon sources could cause problems maintaining stable cultures. Although optimized conditions of several experiments lead to PHA contents of more than 50% of cdw, the total cdw remained mostly under 1 g/L and the overall productivity and growth rate of *Nostoc* is relatively low. Table 3 shows reported PHA values of *Nostoc*.

### 3.4. Other Cyanobacteria

Recently, the PHB content of 137 cyanobacterial strains representing 88 species in 26 genera was determined under photoautotrophic conditions. High PHB content was highly strain-specific and was not associated with the genera. From the 137 tested strains, 134 produced PHB and the highest content was measured in *Calothrix scytonemicola* TISTR 8095 (Thailand Institute of Scientific and Technological Research). This strain produced 356.6 mg/L PHB in 44 days and reached a PHB content of 25% of cdw and a total biomass of 1.4 g/L. The PHB content of 25% was reached under nitrogen depletion, while cells with nitrogen supply reached a PHB content of only 0.4%. From the 19 tested *Calothrix* strains, only six produced more than 5% PHB of cdw. One of the greatest advantages of *Calothrix* is the relative ease of harvesting the dense flocs of algae, but cultivation of *Calothrix* is still at a very early stage [38].

The filamentous diazotroph cyanobacterium *Aulosira fertilissima* produced 10% PHB of cdw under photoautotrophic conditions and phosphate deficiency. The PHB content was boosted to 77% under phosphate deficiency with 0.5% acetate supplementation. This study also shows the positive effect of other carbon sources like citrate, glucose, fructose and maltose on PHB production [67]. Anabaena cylindrica, a filamentous cyanobacterium, was examined for PHB and P[3HB-co-3HV] production. Under nitrogen depletion with acetate supply, *Anabeana cylindrica* produced 2% PHB of cdw and a total biomass of 0.6 g/L. This organism was also able to produce the co-polymer P[3HB-co-3HV] when supplemented with valerate and propionate [68]. Table 4 shows reported PHA values of different cyanobacterial species.

## 4. CO_2_ and Nutrient Supply for Mass Cultivation of Cyanobacteria

### 4.1. CO_2_ Supply

Today, commercial microalgae production is still mainly taking place in open ponds. Here, the C source is normally sodium bicarbonate or atmospheric CO_2_. In order to boost productivities in open systems, or if photobioreactor systems are employed, the use of commercial CO_2_ from gas cylinders is common [70].

However, current production systems are used for the production of high value products (food, feed additives), where CO_2_ price is not critical. If PHA is to be produced, which has a lower economic value, cheap CO_2_ sources are of interest. Although there is considerable literature on various CO_2_-sources (e.g., flue gases) and microalgae growth, there is very limited literature available on cyanobacteria and alternative CO_2_-sources. Table 5 summarizes the literature on cyanobacterial growth on flue gases or fermentation gases.

### 4.2. Nutrient Supply

The cultivation of microalgae and cyanobacteria consume high amounts of nutrients, mainly nitrogen and phosphorous [77]. For research, and even cultivation, mainly synthetic nutrient sources are used [78]. By using alternative nutrient sources, like agro-industrial effluents, waste waters, or anaerobic digestate, questions concerning sustainability of cyanobacteria cultivation, which arise by using fertilizer as a synthetic nutrient source, can be answered [78]. The biomass concentration achieved in open, as well as in closed, cultivation systems are 0.5–1 g/L and 2–9 g/L, respectively [79]. Therefore, large amounts of water are needed. Recycling of process water is another important approach for a more sustainable microalgae cultivation.

In addition to their low costs, the advantages of using alternative nitrogen and phosphorous sources include the production of valuable biomass while removing nutrients from wastewaters, as well as the prevention of competition with food and feed production [78]. On the other hand, new challenges arise, including microbial contaminations, heavy metals and growth inhibitors, suspended solids, or dissolved organic compounds contained in wastewaters, as well as the seasonal composition and fluctuation in amounts [80]. To cope with these challenges recent research focused on cultivating cyanobacteria in anaerobic digestate and agro-industrial effluents or wastewaters for removing nutrients [81,82,83,84,85,86] (see Table 6) and on integrating cultivation processes into biorefinery systems [83].

Additionally, process water and nutrients after harvesting cyanobacterial biomass [79] and product extraction can be directly recycled. Biomass can also be anaerobically digested [87,88] or hydrothermally liquefied via HTL (mineralization of organic nutrients) [89,90] and then recycled. Recycling process water directly can increase the concentration of inhibitory substances and dissolved organic matter from the previous batch produced by cyanobacteria [91], which decrease the productivity of cyanobacteria. Furthermore, nutrient competition may arise by enhanced bacterial growth [79].

Although many publications deal with alternative nutrient sources for cultivating cyanobacteria, hardly any of them focus on cyanobacterial PHA production [66,92]. Reasons for that may be that PHA production requires nutrient limitation [93] and the balance between nutrient limitation, decreased growth and production rates is difficult. The colouring of the nutrient source must be respected as well [94].

## 5. Three Years’ Working Experience Running a Pilot Plant for Photoautotrophic PHB Production

### 5.1. Location and Reactor Description

**Figure 2:**
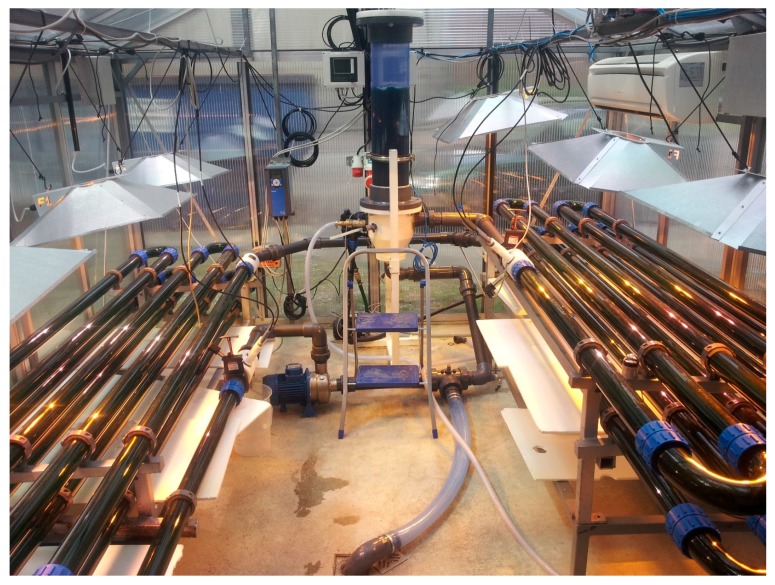
Two-hundred litre tubular photobioreactor with *Synechocystis salina* CCALA192. The central tower serves as a degasser. The centrifugal pump is situated at the lowest point of the reactor on the left side.

The photobioreactor is situated in a glass house at the coal power station in Dürnrohr, Austria. It is a tubular system built from glass elements of Schott AG with an inner diameter of 60 mm, a total length of 80 m and a volume of 200 L (Figure 2). The main design of the photobioreactor is described elsewhere [99,100]. A central degassing unit serves to remove the oxygen as well as to compensate filling level. The medium is circulated with a 400 W centrifugal pump. pH value can be controlled through injection of pure CO_2_ via a mass flow controller. Additional artificial light is provided by six 250 W gas-discharge lamps. Temperature is controlled with an air conditioner.

### 5.2. CO_2_ Supply of the Reactor

The flue gases of the power plant at Dürnrohr usually contain between 11%–13% CO_2_. Next to the chimney is a CO_2_ separation plant (acronym SEPPL), providing the possibility to concentrate the CO_2_ and fill it into gas bottles [101] though, for a more economic approach, the CO_2_ should be used directly without prior compression. The SEPPL provides this option, as well as the possibility to wash the flue gases after the flue gas cleaning of the plant itself to remove residual NO*_x_* and SO*_x_*. Unfortunately for our research project, due to the current situation on the energy market, the power station is no longer run in full operation and only runs occasionally for balancing peak demands of the electrical grid. Therefore, a continuous cultivation with direct utilization of flue gas is not possible. Aspects like this must be respected when planning large industrial cultivation plants.

### 5.3. Automation and pH Control

**Figure 3:**
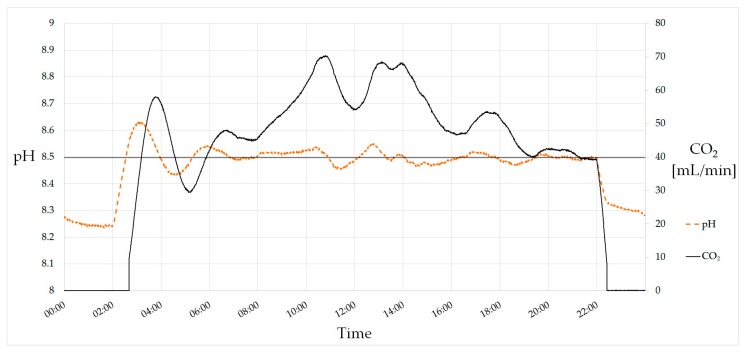
PI-controlled pH value. The setpoint of the pH is 8.5. Lamps turn on at 02:00 and turn off at 22:00, causing a rise and decrease of the pH value due to CO_2_ consumption. In total, 59 L (118 g) of CO_2_ were consumed on this day.

The pH value is one of the most crucial parameters and needs to be controlled carefully. Due to CO_2_ consumption, the pH value rises during photoautotrophic growth. This can be observed when turning on illumination. The tubular photobioreactor is equipped with a PI (Proportional–Integral) controller for pH setting which adjusts the mass-flow controller for CO_2_ inlet. This allows an online control of the currently consumed CO_2_, which is a suitable parameter for growth monitoring. Figure 3 shows a 24-h course of the pH value and the CO_2_ mass flow. Lamps turned on at 02:00 and off at 22:00, causing a rise and decrease of the pH value, respectively. The setpoint of 8.5 is reached after first overshooting and held during the day. The decrease of CO_2_ consumption at noon is caused by the shadow of the power plant’s chimney that casts upon the greenhouse at this time.

### 5.4. Overview of PHB Production Trials

Most of the trials (overview shown in Table 7) were performed using a modified BG 11 medium [102]. Modification in terms of PHB production is necessary, as normal BG 11 medium contains high amounts of nitrogen (1.5 g/L NaNO_3_) and would not lead to nitrogen limitation. The modified BG 11 contains 0.45 g/L NaNO_3_ and leads to a self-limitation of the culture. After 8–12 days nitrogen is consumed, PHB production starts and the color of the culture gradually turns yellow. This approach is necessary, as it is not possible to transfer large-scale cultures into a nitrogen-free medium [103].

*Synechocystis salina* CCALA192 was found to be a very suitable cyanobacterium. It is easy to handle and grows with small inoculation volumes of 1:50. Final biomass and PHB concentrations were in the range of 0.9–2.1 g/L and 4.8% to 9% of cdw, respectively. *Synechocystis salina* CCALA192 also grew with the addition of acetate, but no significant increase of biomass and PHB concentration was observed compared to photoautotrophic growth. When using acetate, contaminations with fungi were likely to occur and trials had to be stopped. Therefore, this approach was finally abandoned.

**Figure 4:**
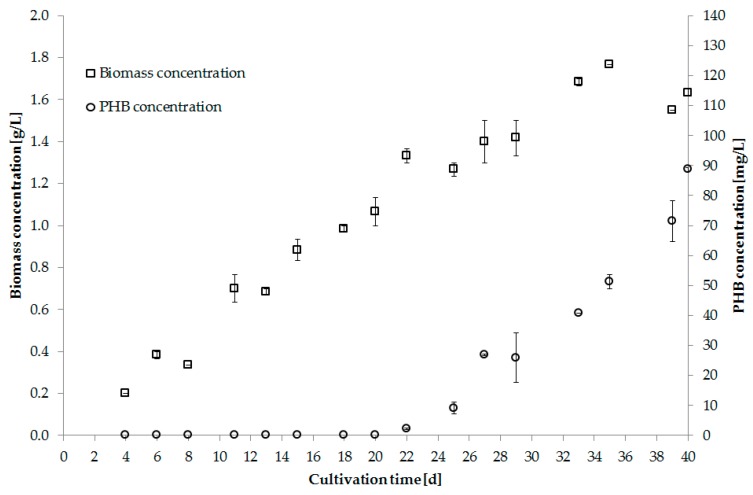
Biomass [g/L] and PHB [g/L] concentration of *Synechocystis salina* using digestate supernatant as nutrient source (Trial 5).

Digestate from a biogas reactor was successfully tested as an alternative nutrient source. The supernatant was produced by centrifugation with prior addition of precipitating agents. Before usage the supernatant was autoclaved and diluted 1:3 with water [92]. Figure 4 shows biomass and PHB production using digestate as nutrient source.

After one and a half years a new degassing system was installed, as the oxygen concentration was mostly above 250% saturation during the day. For an ideal cultivation of cyanobacteria the oxygen saturation should not exceed 200%. The new degasser led to a rise in biomass production with a maximum production rate of 0.25 g/L/d. Efficient degassing affected the cyanobacteria positively. However, during installation of the degasser dirt from the surrounding soil was brought into the reactor and from that time on culture crashes occurred due to ciliate contaminations (see Section 5.6).

The other tested cyanobacteria *Chlorogloeopsis fritschii* and *Arthrospira* sp. could not be successfully cultivated in the photobioreactor. It is assumed that these strains were sensitive to shear stress caused by the centrifugal pump [104].

### 5.5. Downstream Processing of Cyanobacterial Biomass

Downstreaming of cyanobacterial cultures is particularly difficult, as cell densities are much lower compared to heterotrophic bacteria. Typical harvesting methods are sedimentation, filtration, or centrifugation [105]. The cyanobacterial biomass was harvested with a nozzle separator and stored at −20 °C. The biomass was then used to evaluate processing steps necessary to gain clean PHB-samples for quality analysis. These downstream trials include (i) different cell disruption methods (milling, ultrasound, French press); (ii) different pigment extraction methods (with acetone and ethanol/methanol before or after extracting PHB); and (iii) different PHB extraction methods (soxhlet extraction with chloroform, biomass digestion with sodium hypochlorite) [106].

**Figure 5:**
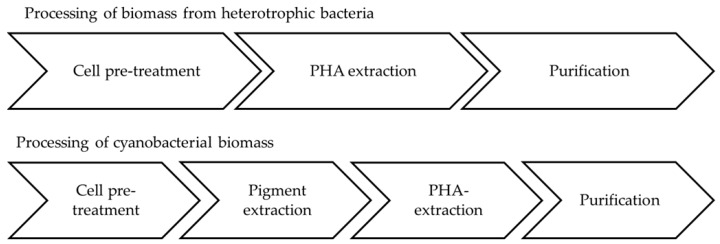
Comparison of processing steps needed to extract PHA from heterotrophic bacteria and cyanobacteria.

These trials showed that cell disruption with French press worked quite well but is very time consuming. Milling is assumed to decrease the molecular weight (polymer chain length). Pigment removal turned out to be necessary prior to PHB extraction, as pigments influenced the PHB properties negatively. This process step can be of advantage due to the generation of phycocyanin as a valuable side product [107]. A mixture of acetone and ethanol (70:30) was most suitable for this purpose. PHB extraction was performed with hot chloroform via a soxhlet extractor. Figure 5 compares the necessary processing steps of heterotrophs and cyanobacteria.

The PHB analysis showed that the polymers extracted from cyanobacterial biomass are comparable to commercially available PHB. Furthermore, it was shown that not only did the nutrient source, but also biomass pre-treatment and the method of polymer extraction influence the PHB properties. Pigment extraction and sample pre-treatment increased the average molecular weight (*M_w_*) from 0.3 to 1.4 MD, but decreased degradation temperatures and crystallinity from 282 °C to 275 °C (*T_onset_*) and from 296 °C to 287 °C (*T_max_*), respectively. The *M_w_* ranged from 5.8 to 8.0 MD, by using mineral medium and digestate, respectively. The thermal properties (*T_onset_*: 283–282 °C; *T_max_*: 293–292 °C), which are important for processing the polymer, are only slightly influenced by the nutrient source and are lower than, but comparable to, commercially available PHB. The crystallinity, responsible for higher final brittleness of the products, is about 17% lower than commercially available PHA.

### 5.6. Contaminations

Contaminations in non-sterile mass cultivation of microalgae are inevitable. It is only a matter of time before first contaminations appear, whether cultivation is done in open ponds or closed photobioreactors [108]. We observed certain bacterial and fungal contaminations with minor effects on *Synechocystis salina* CCALA192, when using CO_2_ as the sole carbon source. Though, when adding acetate to the medium fungal contaminations were prevalent and difficult to control. After one and a half years the pilot plant was revised and a new degassing system was installed. From this moment on rapid culture crashes occurred. The microscopic image revealed a ciliated protozoa ingesting *Synechocystis* rapidly.

This ciliate forms highly resistant cysts and it is assumed that cysts from the surrounding soil were brought into the system during the revision work. Facts about contaminations in mass cultivation of *Synechocystis* are scarcely reported. Touloupakis and colleagues reported the grazing of *Synechocystis* PCC6803 by golden algae *Poterioochromonas* sp. [109]. High pH values of 10 and above helped to control the contaminant and maintain a stable culture. Unfortunately, the ciliate in our cultures survives those high pH values. Thoroughly cleaning and sanitizing the photobioreactor brought some success, but the ciliate is still occurring and leading to culture crashes. Due to the ciliate’s capability to form cysts, it is very difficult to completely eliminate it from the reactor. Heat sterilization is not possible in tubular photobioreactors. Addressing further research, there is need for special cultivation methods for robustly growing *Synechocystis* in non-sterile environment.

## 6. Conclusions

Although not economical today, the idea of a sustainable PHB production with cyanobacteria, CO_2_, and sunlight is still attractive and, more and more, researchers are working in this field. The main challenges today are similar to biofuel production with green algae: (i) realization of efficient low-cost cultivation systems at large scale; (ii) maintaining stable cultures under non-sterile conditions; (iii) increasing the total productivity and yield; and (iv) economic downstream processing and utilization of the residual biomass.

Looking for suitable production strains it must be considered that PHB production is a very common feature of many, but not all, cyanobacteria. The PHB content of cyanobacteria is highly strain specific, as strains of the same genus were reported with highly varying PHB contents. In addition to the PHB content the growth rate and robustness of a strain is particularly important. The only cyanobacterium cultivated in mass cultivation today is *Arthrospira* sp. and, therefore, one of the most promising candidates for photoautotrophic PHB production, although most *Arthrospira* sp. strains still show little PHB content.

Heterotrophic cultivation with acetate boosts the PHB content remarkably, as most reported values over 30% were achieved this way. However, it needs to be considered that using an organic carbon source impairs the most attractive feature of cyanobacteria, converting CO_2_ to PHB. Using organic sources will also complicate non-sterile mass cultivation and could easily lead to contaminations and culture crashes. PHB production with organic carbon sources should be performed with heterotrophic bacteria, as their PHB productivity, as well as their cell density, are 10–100 times higher.

Nitrogen and phosphorous depletion are the most important factors to increase the PHB content and are often necessary to produce any PHB at all. Therefore, a two-stage cultivation with a self-limiting medium is necessary for large-scale photoautotrophic PHB production. With this strategy PHB was successfully produced in our 200 L photobioreactor. In tubular systems small unicellular organisms, like *Synechocystis* sp., are preferred over filamentous organisms, mainly because of the shear stress of the pump. Considering all of the difficulties to overcome, establishing a stable cyanobacterial culture is most important and most difficult to achieve.

## Figures and Tables

**Table 1:** *Synechocystis* and *Synechococcus* as PHA producers. (cdw = cell dry weight, n.r. = not reported).

Carbon Source	Cyanobacterium	Culture Condition	%PHA of cdw	PHA Composition	Total cdw	Reference
Photoautotrophic	*Synechocystis* PCC6803	Photoautotrophic, nitrogen lim.	4.1%	PHB	0.65 g/L	[45]
	*Synechocystis* PCC6803	Photoautotrophic, nitrogen lim.	9.5%	PHB	n.r.	[46]
	*Synechocystis* PCC6803	Photoautotrophic, phosphate lim.	11.2%	PHB	n.r.	[46]
	*Synechocystis* PCC6803 (recombinant)	Photoautotrophic, nitrogen lim.	26%	PHB	n.r.	[47]
	*Synechococcus* MA19	Photoautotrophic, phosphate lim., 50 °C	55%	PHB	4.4 g/L	[48]
Heterotrophic	*Synechocystis* PCC6803	Acetate + Fructose supplementation	38%	PHB	n.r.	[46]
	*Synechocystis* PCC6803 (recombinant)	Acetate supplementation	35%	PHB	n.r.	[47]

**Table 2:** *Arthrospira* as a PHA producer. (cdw = cell dry weight, n.r. = not reported).

Carbon Source	Cyanobacterium	Culture Condition	%PHA of cdw	PHA Composition	Total cdw	Reference
Photoautotrophic	*Arthrospira platensis*	Photoautotrophic	6%	PHB	n.r.	[54]
	*Arthrospira* sp.	Photoautotrophic	<1%	PHB	n.r.	[55]
	*Arthrospira platensis*	Photoautotrophic, phosphate lim.	3.5%	PHB	0.3 g/L	[56]
	*Arthrospira subsalsa*	Photoautotrophic, nitrogen lim.	14.7%	PHB	1.97 g/L	[57]
	*Arthrospira platensis*	n.r.	22%	PHB	n.r.	[59]
Heterotrophic	*Arthrospira maxima*	Acetate + CO_2_	5%	PHB	1.4 g/L	[26]
	*Arthrospira* sp.	Acetate + CO_2_	2.5%	PHB	n.r.	[55]

**Table 3:** *Nostoc* as a PHA producer. (cdw = cell dry weight, n.r. = not reported).

Carbon Source	Cyanobacterium	Culture Condition	%PHA of cdw	PHA Composition	Total cdw	Reference
Photoautotrophic	*Nostoc muscorum*	Photoautotrophic, nitrogen and phosphorous lim.	8.7%	PHB	n.r.	[62]
	*Nostoc muscorum agardh*	Photoautotrophic, 10% CO_2_	22%	PHB	1.1 g/L	[66]
	*Nostoc muscorum*	Photoautotrophic, nitrogen and phosphorous lim.	22%	PHB	0.13 g/L	[56]
Heterotrophic	*Nostoc muscorum agardh*	Acetate, valerate, nitrogen lim.	58%	P[3HB-co-3HV]	0.29 g/L	[64]
	*Nostoc muscorum*	Acetate, limited gas exchange	40%	PHB	n.r.	[63]
	*Nostoc muscorum agardh*	Acetate, glucose, valerate, 10% CO_2_	70%	P[3HB-co-3HV]	0.98 g/L	[66]
	*Nostoc muscorum agardh*	Acetate, glucose, valerate, nitrogen lim.	78%	P[3HB-co-3HV]	0.56 g/L	[65]
	*Nostoc muscorum*	Acetate, dark incubation, nitrogen and phosphorous lim.	35%	PHB	n.r.	[62]

**Table 4:** Different cyanobacterial species as PHA producers. (cdw = cell dry weight, n.r. = not reported).

Carbon Source	Cyanobacterium	Culture Condition	%PHA of cdw	PHA Composition	Total cdw	Reference
Photoautotrophic	*Phormidium* sp. TISTR 8462	Photoautotrophic, nitrogen lim.	14.8%	PHB	n.r.	[38]
	*Oscillatoria jasorvensis* TISTR 8980	Photoautotrophic, nitrogen lim.	15.7%	PHB	n.r.	[38]
	*Calothrix scytonemicola* TISTR 8095	Photoautotrophic, nitrogen lim.	25.2%	PHB	n.r.	[38]
	*Anabaena* sp.	Photoautotrophic	2.3%	PHB	n.r.	[69]
	*Aulosira fertilissima*	Photoautotrophic, phosphorous lim.	10%	PHB	n.r.	[67]
Heterotrophic	*Aulosira fertilissima*	Acetate, phosphorous lim.	77%	PHB	n.r.	[67]
	*Aulosira fertilissima*	Maltose, balanced	15.9%	PHB	2.3 g/L	[67]

**Table 5:** Growing cyanobacteria with alternative CO_2_-sources.

Type of Gas	Cyanobacterium	CO_2_ Source	Reference
Flue gases	*Phormidium valderianum*	Coal combustion flue gas	[71]
	*Atrhrospira platensis*	Coal combustion flue gas	[72]
	*Arthrospira* sp.	Synthetic flue gas	[73]
	*Synechocystis* sp.	Flue gas from natural gas combustion	[74]
CO_2_ rich fermentation gases	*Arthrospira platensis*	CO_2_-offgas from ethanol fermentation	[75]
	*Arthrospira platensis*	Biogas	[76]

**Table 6:** Overview of agro-industrial effluents and wastewaters and anaerobic digestates used as nutrient sources for cultivating cyanobacteria.

Nutrient Source		Cyanobacterium	Total cdw/Growth Rate	Product/Purpose	Reference
Agro-industrial effluents and waste waters	Raw cow manure	*Arthrospira maxima*	3.15 g/L	Biomass production	[80]
	Molasses	*Arthrospira platensis*	2.9 g/L	Biomass production	[95]
	Olive-oil mill wastewater	*Arthrospira platensis*	1.69 g/L	Nutrient removal	[84]
	Poultry litter	*Nostoc muscorum agardh*	0.62 g/L	PHA production	[66]
Anaerobic digestate	Waste from pig farm	*Arthrospira platensis*	20 g/m^2^/d	Nutrient removal	[81]
	Digested sago effluent	*Arthrospira platensis*	0.52–0.61 g/L	Nutrient removal	[96]
	Digestate from municipal solid waste	*Arthrospira platensis*	Growth rate 0.04 d^−1^	Nutrient removal	[97]
	Digestate from vegetable waste	*Arthrospira platensis*	Growth rate 0.20 d^−1^	Nutrient removal	[97]
	Waste from pig farm	*Arthrospira* sp.	15 g/m^2^/d	Nutrient removal	[85]
	Algal digestate	*Chroococcus* sp.	0.79 g/L	Nutrient removal	[86]
	Digestate sludges	*Lyngbya aestuarii*	0.28 g/L	Biomass production	[83]
	Digestates of *Scenedesmus* spp.	*Lyngbya aestuarii*	0.11 g/L	Biomass production	[83]
	Thin stillage digestate	*Synechocystis* cf. *salina Wislouch*	1.6 g/L	PHB production	[92]
	Anaerobic digester effluent	*Synechocystis* sp.	0.15 g/L	Lipid production	[98]

**Table 7:** Overview of selected trials conducted in a tubular photobioreactor at pilot scale.

Trial	Strain	Nutrient Solution	Cultivation Time	Final Biomass Concentration	Final PHB-Concentration of cdw
1. Mineral medium	*Synechocystis salina* CCALA192	Optimized BG11	June 21 days	2.0 ± 0.12 g/L	6.6% ± 0.5%
2. Acetate addition	*Synechocystis salina* CCALA192	Optimized BG11, 20 mM acetate	July 26 days	1.9 ± 0.02 g/L	6.0% ± 0.1%
3. Acetate addition	*Synechocystis salina* CCALA192	Optimized BG11, 60 mM acetate	September 24 days	Trial cancelled, due to contaminations with fungi	
4. 24 h illumination	*Synechocystis salina* CCALA192	Optimized BG11	October 27 days	1.8 ± 0.02 g/L	4.8% ± 0.0%
5. Alternative nutrient source	*Synechocystis salina* CCALA192	Digestate supernatant	November–December 40 days	1.6 ± 0.02 g/L	5.5% ± 0.3%
6. Mineral medium	*Synechocystis salina* CCALA192	Optimized BG11	December–January 30 days	2.1 ± 0.03 g/L	6.0% ± 0.02%
7. Optimal degassing	*Synechocystis salina* CCALA192	Optimized BG11	May 7 days	0.9 ± 0.03 g/L (Trial prematurely cancelled due to ciliates)	9% ± 0.1% (Trial prematurely cancelled due to ciliates)
8. Chlorogloeopsis fritschii CCALA39	*Chlorogloeopsis fritschii* CCALA39	Optimized BG11	February 11 days	Trial cancelled, due to lack of growth	
9. Arthrospira	*Arthrospira* sp.	Spirulina Medium	October 7 days	Trial cancelled, due to lack of growth	
